# Analysis of risk factors affecting mortality in elderly patients operated on for hip fractures: A retrospective comparative study

**DOI:** 10.5152/j.aott.2021.21004

**Published:** 2021-11-01

**Authors:** Çağdaş Biçen, Mehmet Akdemir, Mehmet Aykut Türken, Kübra Çekok, Ahmet Ekin, Ahmet Cemil Turan

**Affiliations:** 1 Department of Orthopedics and Traumatology, İzmir University of Economics, Medical Park Hospital, İzmir, Turkey; 2 Clinic of Orthopedics and Traumatology, İzmir Ekol Hospital, İzmir, Turkey; 3 Department of Physical Medicine and Rehabilitation, İzmir University of Economics, Medical Park Hospital, İzmir, Turkey

**Keywords:** Hip fracture, Mortality, Elderly, Cementless, Nail, Proximal femoral nail

## Abstract

**Objective:**

The aims of this study were (1) to investigate the effects of different demographic and perioperative modalities on mortality rates and (2) to compare mortality rates between different implants in elderly patients operated on for hip fractures.

**Methods:**

In this retrospective study, a total of 314 patients who were operated on for hip fractures were included study. Patients were then divided into four groups based in their implant types: long-stem cementless bipolar hemiarthroplasty (n = 124; 102 female, 22 male; mean age = 84.2 ± 6.4 years), standard-stem cementless bipolar hemiarthroplasty (n = 74; 48 female, 26 male; mean age = 83.5 ± 6.9 years), antegrade intertrochanteric nail (n = 61; 35 female, 26 male; mean age = 78.5 ± 6.8 years), and total hip arthroplasty (n = 55; 34 female, 21 male; mean age = 72.5 ± 4.3 years). Data including gender, age, duration from injury to surgery, American Society of Anesthesiologists (ASA) score, comorbidities, use of antiplatelet agents, Barthel Index of Activities of Daily Living, type of anesthesia, operation time, preoperative hemoglobin values, blood transfusions given, duration of hospital stay, complications, and type of fracture were recorded.

**Results:**

Overall, the mean follow-up was 36.5 (range = 0 – 107) months. The overall mortality rate was 53.2%. The median survival duration was 44.2 ± 5 months (range = 34.3 – 54). Survival rates were found significantly different among the groups (*P* = 0.001). In the first three years postoperatively, the mortality rate was higher in the standard-stem bipolar hemiarthroplasty group, but in the long-term follow-up, the long-stem bipolar hemiarthroplasty group exhibited the higher mortality rates. It was observed that some parameters had statistically significant effects on the mortality rates. Male gender, higher age, lower hemoglobin values, increased number of blood transfusions, ASA scores ≥3, the existence of ≥ 3 comorbidities were found as main predictors of increased mortality rates.

**Conclusion:**

The results of this study have shown that age, gender, preoperative hemoglobin levels, ASA scores, and comorbidities are significant factors affecting mortality in elderly patients operated on for hip fractures. Long-stem cementless bipolar hemiarthroplasty appears to show similar rates of mortality with standard-stem cementless bipolar hemiarthroplasty.

**Level of Evidence:**

Level III, Therapeutic Study

## Introduction

Hip fractures are among common problems in the elderly and can be considered as a public health issue.^1^ Increased mortality has been reported in elderly patients after hip fractures, and these rates are known to be significantly high when compared with individuals of similar age in the general population.^2-^^7^ Factors increasing mortality rates following surgical treatment of hip fractures in the elderly are still not clear.^8^ There are two main types of hip fractures, which are intracapsular and extracapsular fractures. Different surgical procedures have been defined for these fracture types. The mobility and functional capacities of patients with hip fractures have been assessed between different fracture types and implants. Moreover, the functional scores and complications have been compared between fixation and hemiarthroplasty, and there have also been studies that have compared the outcomes of hemiarthroplasty and total hip arthroplasty (THA).^9,10^ The factors affecting mortality rates in elderly patients operated on for hip fractures were also evaluated in the literature; however, studies comparing the mortality rates of different types of implants are limited. In this study, it was aimed to evaluate the relations between mortality and different factors, and demographic and perioperative modalities in elderly patients operated on for hip fractures. Additionally, the effects of the different implants used on the mortality rates were investigated.

## Materials and Methods

**Figure 1.**
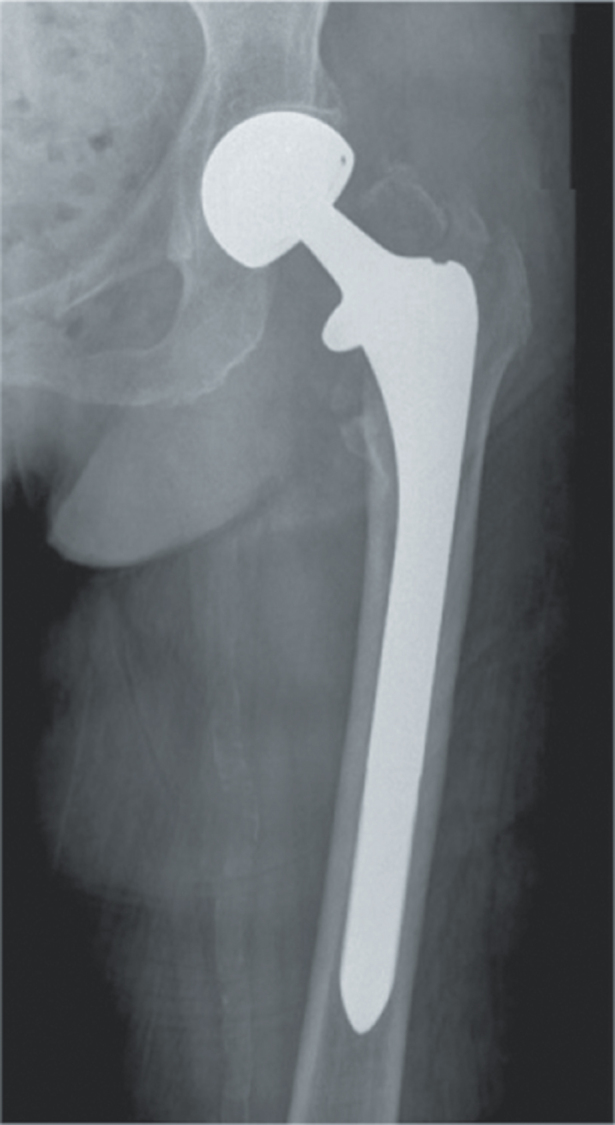
X-Ray views of the long-stem cementless bipolar hemiarthroplasty (LSCBH).

**Figure 2.**
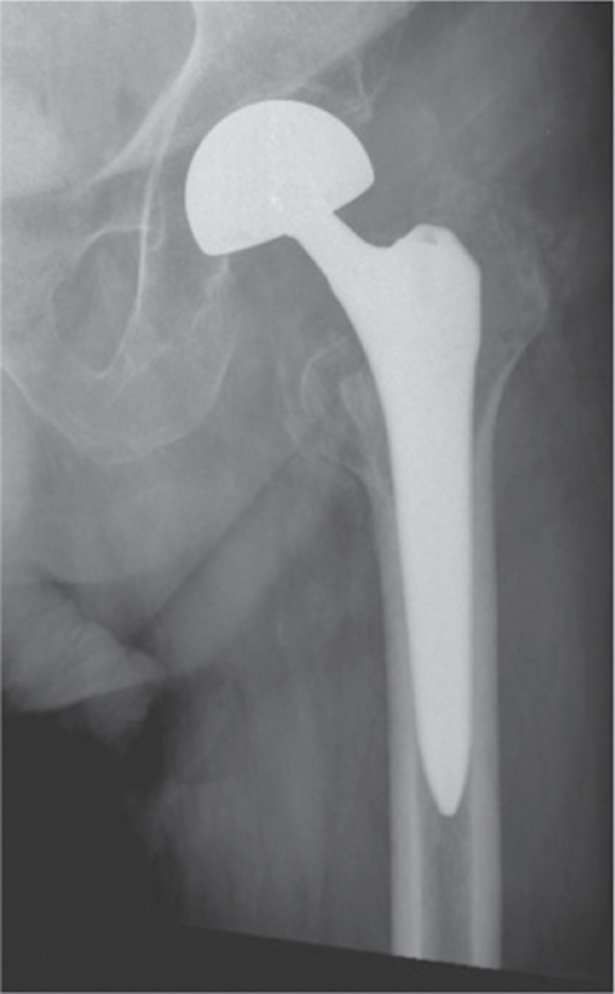
X-Ray view of the standard-stem cementless bipolar hemiarthroplasty (SSCBH).

**Figure 3.**
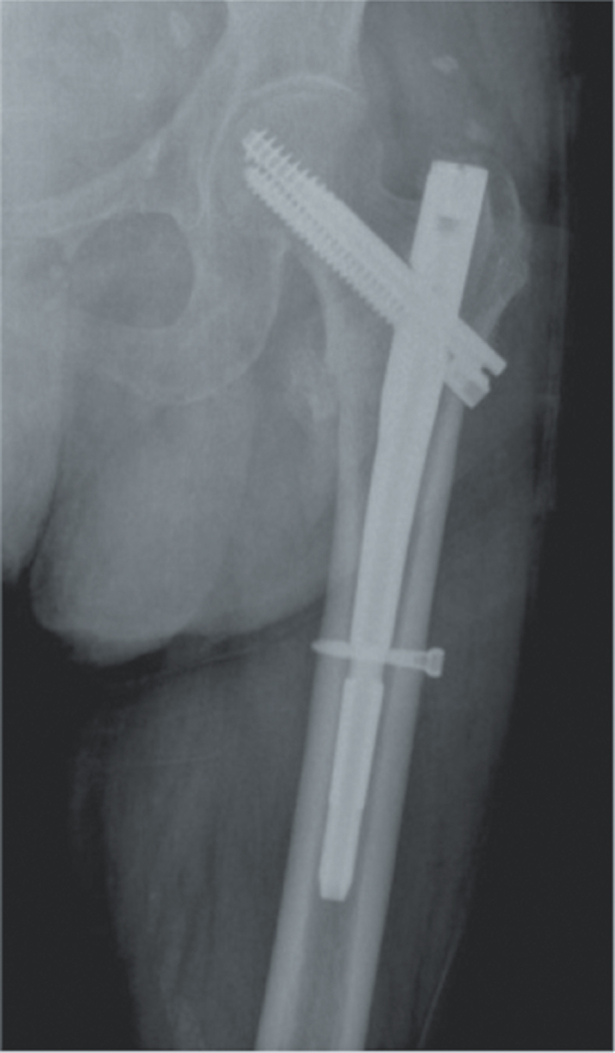
X-Ray view of the antegrade intertrochanteric nail (AIN).

**Figure 4.**
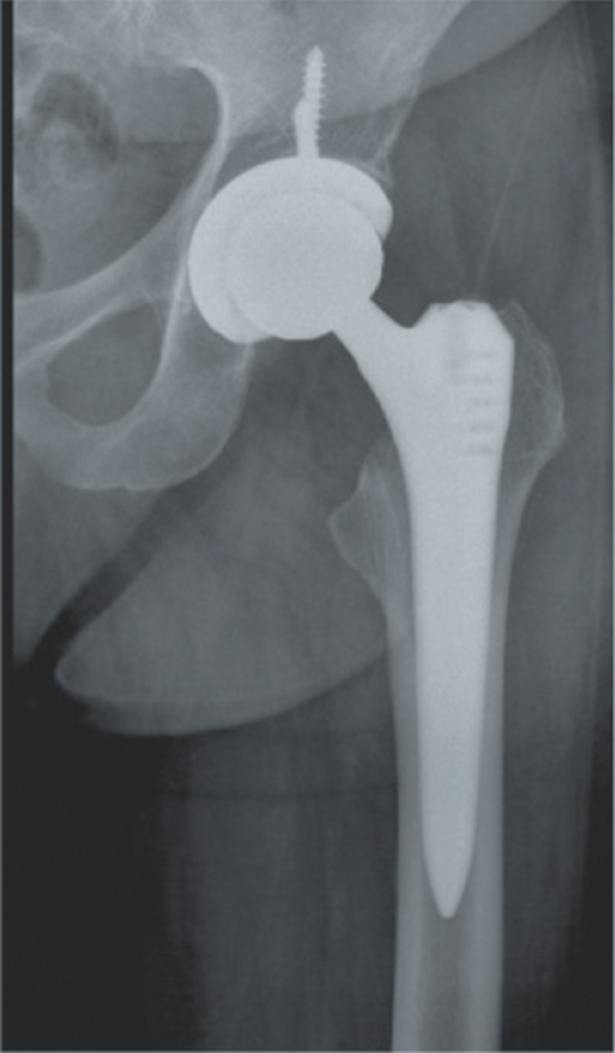
X-Ray view of the total hip arthroplasty (THA).

Retrospectively evaluated were 314 patients who had been surgically treated for hip fractures in our hospital between November 2011 and February 2020. Patients ≥ 65 years of age, who had been operated on with antegrade intertrochanteric nail (AIN) (Trigen InterTan, Smith & Nephew, Memphis, TN, USA), long-stem cementless bipolar hemiarthroplasty (LSCBH) (Echelon, Smith & Nephew, Memphis, TN, USA), standard-stem cementless bipolar hemiarthroplasty (SSCBH), or cementless THA (SL-PLUS, Smith & Nephew, Memphis, TN, USA), due to collum femoris or intertrochanteric fractures were included in the study (Figures 1-4). Patients with accompanying pelvic and/or other fractures, bilateral hip fractures, pathological fractures, those who had been bedridden before the fracture, and those with missing medical records in their hospital files were excluded from the study. Data regarding gender, age, duration from injury to operation (day), American Society of Anesthesiologists (ASA) score, comorbidities, use of antiplatelet agents, Barthel Index of Activities of Daily Living (BIADL) SCORE, type of anesthesia, operation time (minute), preoperative hemoglobin values (g/L), red blood cell transfusions given (unit), duration of hospital stay (day), complications, type of fracture, and type of implant were collected. Moreover, the time from surgery to death or time of follow-up until the cut-off date of the study (month) were recorded.

The long-stem prosthesis was 190 or 220 mm long, made of cobalt-chromium, and porous-coated. . The standard-stem prosthesis was a straight stem with a rectangular cross-section, with length options of 128-188 mm, made of cobalt-chromium, and coated with porous. The nail was 18 or 20 cm long, with neck angle options of 125° or 130°. The intertrochanteric fractures were accepted as extracapsular fractures of the proximal femoral located between the lesser and greater trochanter, while the femoral neck fractures were accepted as intracapsular fractures of the proximal femur located in the connection of the femoral head with the femoral shaft. Surgical methods were chosen based on the age of the patient, type of fracture, the comminution of the fracture, comorbidities, and prefracture functional status of the patients. Stable intertrochanteric fractures were operated on with AIN, femoral neck fractures, and unstable intertrochanteric fractures were operated on with arthroplasty. The type of arthroplasty was chosen considering the cognitive status, functional status, and tolerability of anesthesia. Patients who could walk outdoors using walking sticks or a walker, those with cognitive disorders, and those with lower tolerability of anesthesia were operated on with hemiarthroplasty. The length of the stem was chosen based on the prefracture diagnosis of osteoporosis. For patients who had a diagnosis and treatment for osteoporosis, a long-stem prosthesis was chosen. There were no major changes in the implants used, and the implant preference was not changed for the same fracture types during the study period. All of the operations were performed by the same surgical team under spinal or general anesthesia. Positioning was lateral decubitus in all of the operations, and the lateral approach was used for all of the arthroplasties. Isometric and quadriceps strengthening exercises were begun on the first day postoperatively after all of the surgeries. Patients who underwent arthroplasty were encouraged to perform full weight-bearing mobilization on the first day postoperatively. The patients operated on with AIN were mobilized without weight-bearing mobilization on the first day postoperatively and weight-bearing mobilization was increased gradually. Full weight-bearing mobilization was allowed approximately 4 months postoperatively for these patients. Postoperative follow-up was performed at 6 weeks, and 3, 6, and 12 months postoperatively, and every year thereafter. The Arbeitsgemeinschaft für Osteosynthesefragen (AO) classification was used to classify the fractures. The BIADL was used to determine the prefracture functional capacity.^11^ Comorbidities, the use of antiplatelet agents, and the ASA scores were recorded from the patient files of the preoperative examinations. An ASA score of 1 or 2 was defined as low risk, while an ASA score ≥ 3 was defined as high risk.^12^ Comorbidities were assessed in a number of accompanying systemic diseases, as < 3 or ≥ 3,^13^ which included hypertension, diabetes, cardiac disorders, dementia, malignancy, and endocrinological and neurological diseases. Blood transfusion was performed in patients who had a hemoglobin level < 85 g/L and in those with complaints and symptoms due to anemia. Complications were recorded as early postoperative and late complications. Ethics committee approval was received for this study from the Medical Research Ethics committee of our institute (Institutional ethical approval no: B.30.2.İEÜSB.0.05-20-102).

## Statistical analysis

The data of the patients were loaded into the Microsoft Excel program. Statistical analysis was performed using IBM SPSS Statistics for Windows 18.0 (IBM SPSS Corp., Armonk, NY, USA). To evaluate the numerical data, the mean ± standard deviation, median (minimum, maximum) were used, while for the categorical data, the number and percentage were used. The distribution of the discrete and continuous numerical variables was analyzed using the Kolmogorov–Smirnov test. The relationship between the categorical data was analyzed using the chi-square test. In cases in which numerical data were normally distributed, 1-way analysis of variance (ANOVA) was used for multiple group comparisons, and the Kruskal–Wallis test was used when the data were not normally distributed. After identifying differences between the groups using the Kruskal–Wallis tests, which groups were different was determined using the Dunn-Bonferroni post hoc method. Survival rates were calculated using Kaplan-Meier survival analysis. The effects of the performed surgery on the survival rates were analyzed using the Log-rank test. In the multivariate analysis, independent factors in predicting the survival rates were analyzed using Cox regression analysis with the simultaneous (enter) selection method using possible factors that were determined to be effective on the survival rates in previous analyses. Among the interrelated parameters showing similar effects on survival, those that were clinically significant were selected for the model. *P* < 0.05 was considered statistically significant.

## Results

The mean age of the patients was 81 (65-101) years, and 95 (30.3%) were male, while 219 (69.7%) were female. Of the patients, 163 (51.9%) had collum femoris fractures, and 151 (48.1%) had intertrochanteric fractures. Moreover, 124 (39.5%) patients were treated with LSCBH, 74 (23.6%) were treated with SSCBH, 55 (17.5%) were treated with THA, and 61 (19.4%) were treated with AIN. The mean time duration between injury and operation was 2 days. The average ASA score was 3 [113 (36%) patients < 2; 201 (64%) patients ≥ 3]. Comorbidities did not exist, or there were less than three comorbidities present in 236 (75.2%) of the patients, and there were three or more comorbidities present in 78 (24.8%) of the patients. Moreover, 58 (18.5%) patients had been using antiplatelet agents before surgery. The average BIADL score of the patients was 82. Additionally, 57 (18.2%) of the patients were operated on under general anesthesia, and 257 (81.8%) of the patients were operated on under regional anesthesia. The average operation time was 81 (±29.35) min. The mean units of red blood cells transfused was 2 per patient. The mean duration of hospital stay was 6 (±2.06) days (Table 1).

Between the groups, a statistically significant difference was found between age, gender, discrimination of fractures, duration from injury to surgery, duration of hospital stay, operation time, BIADL score, preoperative hemoglobin values, and blood transfusion (*P* < 0.05). There was no statistically significant difference between the groups with regard to the ASA scores and comorbidities (*P* = 0.15, *P* = 0.34). Moreover, the use of antiplatelet agents and type of anesthesia were similar between the groups.

The ages of the patients were statistically higher in the LSCBH and SSCBH groups when compared to the AIN group (*P* < 0.001, *P* < 0.001). The ages of the patients were lower in the THA group than in the AIN group (*P* < 0.001). The time from injury to surgery was higher in the THA group when compared with the LSCBH group (*P* < 0.001). The duration of hospital stay was statistically higher in the THA, LSCBH, and SSCBH groups when compared to the AIN group (*P* = 0.012, *P* < 0.001, *P* < 0.001). The operation time was higher in the AIN and THA groups than in the LSCBH and SSCBH groups (*P* < 0.001). The BIADL score of the THA group was statistically significantly higher than in the AIN, LSCBH, and SSCBH groups (*P* < 0.001). The preoperative hemoglobin values were significantly higher in the THA group than in the LSCBH and AIN groups (*P* = 0.003, *P* = 0.021). The necessity of transfusion was higher in the LSCBH group when compared with the AIN group (*P* = 0.006). In the regression analysis, a negative correlation was found between the preoperative hemoglobin values and the need for transfusion.

It was found that some parameters had statistically significant effects on the mortality rates (Table 2). The mortality rates of the female patients were lower than those of the male patients (at 0.67). Age was one of the factors that increased the risk of mortality (at 1.03). Higher hemoglobin values seemed to be among the protective parameters (at 0.93). Controversially, an increased number of blood transfusions raised the rate of mortality (at 1.07). It was observed that increased surgical duration decreased the mortality rates (at 0.9). This rate was due to the longer surgical duration that it took to perform THA by the surgeons at the clinic. Patients with ASA scores ≥ 3 had higher mortality rates when compared to those who had ASA scores < 3 (at a ratio of 1.83, 95% CI = 1.26-2.65). Moreover, the existence of ≥ 3 comorbidities statistically increased the mortality rates (at a ratio of 1.64, 95% CI: 1.14-2.36).

Complications developed in 15 patients. 8 patients suffered from early complications, and 7 patients suffered from late complications. As early complications, 2 patients who underwent THA had a dislocation. Closed reduction was performed in both patients, but one underwent early revision surgery. One patient had a wound infection and healed with debridement. One patient had a urinary tract infection and 1 patient had pneumonia. One patient had popliteal artery thrombosis and 1 patient had below knee thrombosis. One patient who had a periprosthetic fracture before discharge was followed up conservatively. As late complications, 2 patients underwent implant removal due to infection, 3 patients suffered from periprosthetic fractures, 2 underwent plate fixation, and 1 was treated conservatively. Moreover, 2 patients with THA had late dislocation and underwent revision surgery.

The mean duration of follow-up was 36.5 (range: 0-107.2) months. The mean duration of follow-up was 38.5 months in the LSCBH group, 38.6 months in the AIN group, 25.9 months in the SSCBH group, and 43.9 months in the THA group. The overall mortality rate was 53.2% (167 patients). Of the patients, 22 (7%) died within 1 month postoperatively, including the patients who died before discharge; 40.1% of the deaths (67 patients, 21.3%) occurred during the first postoperative year, 21% of the deaths (35 patients, 11.1%) occurred during the second postoperative year, 15.6% of the deaths (26 patients, 8.3%) occurred during the third postoperative year, and 23.4% of the deaths (39 patients, 12.4%) occurred after the end of the third postoperative year (37.7-92.7 months). The median survival duration was 44.2 ± 5 (95% CI: 34.3-54) months. Although it seemed that the mortality rates postoperatively did vary over the total study period, it is accepted that independent predictive factors had a role in affecting the mortality rates at 1 month, 1 year, 2 years, 3 years, and after.

**Figure 5.**
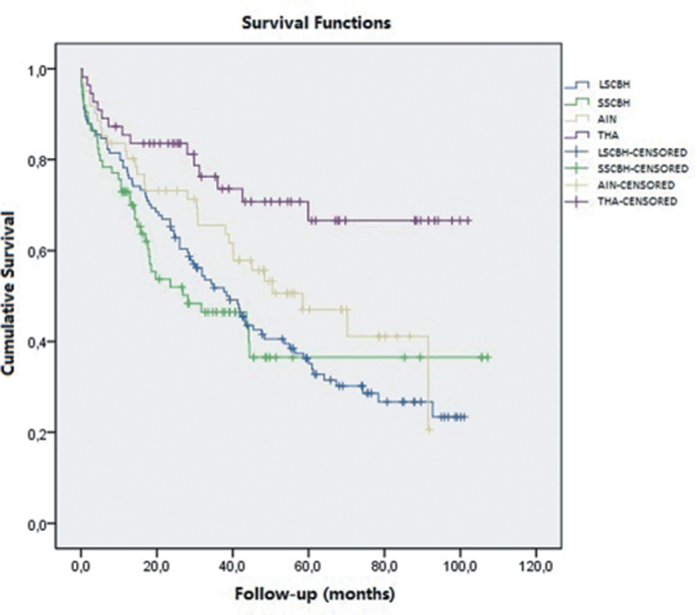
Cumulative survival rates of the groups.

The survival rates were different between the groups (*P* = 0.001). The survival rate of the THA group was significantly higher than those of the LSCBH and SSCBH groups (*P* < 0.001, *P* = 0.06). The survival rate of the AIN group was lower than that of the THA group, but the difference was not statistically significant (Figure 5). In the first three years postoperatively, the mortality rate was highest in the SSCBH group, but in the long-term follow-up, the LSCBH group exhibited the highest mortality rate (Table 3). Of the patients treated with LSCBH, 66.9% died during the follow-up period. This rate was 52.7% for the SSCBH group, 49.2% for the AIN group, and 27.3% for the THA group.

## Discussion

Approximately 1.6 million patients are affected from osteoporotic hip fractures annually, and it is estimated that the global number of hip fractures will increase to 4.5 million by the year 2050, and in Europe, the total direct cost of osteoporotic fractures is expected to be USD$93.2 billion.^14^ Due to the aging of the population, hip fractures are predicted to increase burdens on the economy, and social and health care services.^15^ Improvements in operation methods and perioperative management affected surgical outcomes positively.^16^ However, it was shown in some studies that the rates of mortality remained stable over the years.^17^ A great number of studies have been conducted in order to identify the risk factors affecting mortality rates. However, the issues are not clear enough to prevent high rates of mortality. In the current study, it was aimed to investigate the risk factors that affect mortality rates and compare the mortality rates of four different types of implants.

It was observed in the present study that the duration of hospital stay, time from injury to surgery, use of antiplatelet agents, type of anesthesia, type of fracture, and BIADL scores had no significant effect on the mortality rates in the elderly patients who were operated on due to hip fracture. Gender, age, preoperative hemoglobin values, amount of blood transfused, ASA scores, the existence of comorbidities, and operation time seemed to have had a significant influence on the mortality rates in these patients.

Overall, the mortality rate herein was 53.2%, with a mean follow-up of 36.5 (range: 0-107.2) months. At the end of the first postoperative month, 7% of the patients were deceased. This was followed by 21.3% of the patients at the end of the first postoperative year, 32.4% at the end of the second postoperative year, and 40.7% at the end of the third postoperative year. In accordance with the literature studies, the 1-year mortality rates were between 20% and 32% following hip fracture, and it was reported that the risk of mortality may persist beyond 5 years.^18,19^ In a study conducted in the Netherlands, the overall mortality rates were given as 28% for the first year, 39% for the second year, and 49% for the third year.^4^

Aging was accepted as a risk factor that increased mortality rates in many studies.^20–22^ It was also found in the current study that aging increased the risk of mortality, at a rate of 1.03. It was observed herein that the risk of mortality was higher among male patients. It was proven in the literature that males presented with a higher risk of mortality.^17^ Talsnes et al. evaluated in their study the clinical and biochemical predictive factors of early mortality following surgery due to hip fractures in elderly patients.^23^ They reported that increased age and male gender were correlated with mortality.

In this study, it was observed that the mortality rate was higher among the patients with ASA scores ≥ 3 (at a rate of 1.64, 95% CI: 1.14-2.36). In the study of Aslan et al.,^24^ high ASA scores were shown as an indicator of mortality. Although the ASA scores were seen to have effect on mortality, they were not accepted among the major predictors in the study of Çamurcu et al.^13^

Another factor that was significant was comorbidities, as in many other studies.^25,26^ Of the patients in the current study, 84% had hypertension, diabetes, cardiac disorders, dementia, malignancies, endocrinological, or neurological diseases preoperatively. It was observed that the mortality rates were higher in the patients who had ≥ 3 comorbidities.

The effects of preoperative hemoglobin levels and postoperative transfusions were analyzed previously in the literature, but a common consensus is still not available.^18,27^ In the current study, the protective effect of higher preoperative hemoglobin levels on mortality was observed. Correspondingly, the need for transfusion had a negative effect on the rates.

The difference between the mortality rates regarding the four different implant types were assessed. The highest mortality rate was found in the LSCBH group. The mortality rate of the SSCBH group was similar. The mortality rate was lowest in the THA group and was similar to that of the AIN group. There was a statistically significant difference between the SSCBH and LSCBH groups and the THA group, and there were differences in some parameters between the groups. It was believed that the difference of age, preoperative hemoglobin values, and need for transfusion were responsible for the differences in the mortality rates between the LSCBH and THA groups. Although the demographic data and pre-fracture functional status were not homogeneous between the groups, the results could not be generalized. It was observed that the patients who underwent hemiarthroplasty were older and had poorer pre-fracture functional status. However, in a comparison of the LSCBH and SSCBH groups, which had similar demographic, perioperative, and pre-fracture modalities, the mortality rate in the LSCBH group was not statistically different from that in the SSCBH group.

This study did have limitations, the first of which was its retrospective design. The second limitation was the small number of patients. The choice of implant selection was among the major limitations of the study. The type of surgery was decided by the surgeon, considering the baseline health status and fracture type. Another limitation of the study was the relatively short-term follow-up. Prospectively designed studies and results of studies with longer follow-up will be more informative in this field.

According to the findings of this study, LSCBH seemed to show mortality rates that were similar to SSCBH. Younger age, female gender, and higher preoperative hemoglobin levels were protective factors for mortality in hip fractures in the elderly. Higher ASA scores and the existence of more than two comorbidities were related with increased mortality rates.

**HIGHLIGHTS**

- Age, gender, preoperative hemoglobin levels, ASA scores, and comorbidities were thought to be effective on mortality in elderly patients with hip fractures.
- Younger age, female gender, and higher preoperative hemoglobin levels seemed to have protective effects on mortality in hip fractures in the elderly.
- Mortality rates were statistically similar between LSCBH and SSCBH.

## Figures and Tables

**Table 1.** Demographics and Clinical Data of the Groups

Groups	LSCBH (n = 124)	SSCBH (n = 74)	AIN (n = 61)	THA (n = 55)
Mean age (year), (mean ± SD)	84.2 ± 6.4	83.5 ± 6.9	78.5 ± 6.8	72.5 ± 4.3
Gender n (%)				
Female	102 (82.3)	48 (64.9)	35 (57.4)	34 (61.8)
Male	22 (17.7)	26 (35.1)	26 (42.6)	21 (38.2)
Time from injury to surgery (day), (mean ± SD)	1.81 ± 2.7	2.5 ± 3.6	2.7 ± 2.8	2.3 ± 1.4
Duration of hospital stay (days), (mean ± SD)	5.9 ± 1.8	6.5 ± 1.8	5.3 ± 1.9	6.5 ± 2.9
Preoperative hemoglobin values (g/L) (mean ± SD)	11.1 ± 1.9	11.5 ± 2	11.2 ± 2	12.2 ± 1.8
Operation time (min), (mean ± SD)	70.6 ± 22.8	72.1 ± 23.9	92.2 ± 36.2	101.6 ± 25.6
BIADL score (mean ± SD)	77.6 ± 13.6	77.6 ± 14.8	84.8 ± 13.5	94.6 ± 10.1
ASA score (<3, ≥ 3), n	40/84	23/51	23/38	27/28

BIADL: Barthel Index of Activities of Daily Living, ASA: American Society of Anesthesiologists. LSCBH: long-stem cementless bipolar hemiarthroplasty, SSCBH: standard-stem cementless bipolar hemiarthroplasty, AIN: antegrade intertrochanteric nail, THA: total hip arthroplasty

**Table 2.** Cox Regression Analysis Performed for Evaluating the Factors Affecting the Mortality Rate

	B	SE	Sig.	Exp (B)	95.0% CI for Exp (B)	
					Lower	Upper
Gender	–0.412	0.175	**0.019**	**0.662**	**0.470**	**0.933**
Age (year)	0.031	0.014	**0.020**	**1.032**	**1.005**	**1.060**
Use of antiplatelet agents (yes/no)	0.322	0.217	0.138	1.379	0.902	2.110
Type of fracture (neck/intertrochanteric)	–0.124	0.173	0.475	0.884	0.630	1.240
Duration from injury to surgery (day)	0.040	0.024	0.095	1.041	0.993	1.092
Duration of hospital stay (days)	–0.011	0.046	0.816	0.989	0.905	1.082
Preoperative hemoglobin levels (g/L)	–0.095	0.043	**0.027**	**0.909**	**0.836**	**0.989**
Red blood cell transfusion (unit)	0.074	0.037	**0.048**	**1.077**	**1.001**	**1.159**
BIADL score	–0003	0.006	0.652	0.997	0.986	1.009
Type of anesthesia (general/regional)	0.101	0.219	0.643	1.107	0.721	1.699
Operation time (min)	–0.008	0.003	**0.017**	**0.992**	**0.985**	**0.999**
ASA score (<3, ≥ 3)	0.590	0.188	**0.002**	**1.804**	**1.248**	**2.608**
Comorbidities (<3, ≥ 3)	0.495	0.190	**0.009**	**1.641**	**1.131**	**2.380**
						

Bold values indicate statistically significant. BIADL score: Barthel Index of Activities of Daily Living score

**Table 3.** Cumulative Survival Rates at the End of the Postoperative First, Second, and Third Year

Cumulative survival rates (months)	LSCBH	SSCBH	AIN	THA
1^st^ year	78.2 ± 3.7	73.0 ± 5.2	80.2 ± 5.1	85.4 ± 4.8
2^nd^ year	64.5 ± 4.3	52.0 ± 6.2	73.2 ± 5.7	83.6 ± 5.0
3^rd^ year	51.8 ± 4.5	46.5 ± 6.3	65.6 ± 6.3	73.6 ± 6.4

LSCBH: long-stem cementless bipolar hemiarthroplasty, SSCBH: standard-stem cementless bipolar hemiarthroplasty, AIN: antegrade intertrochanteric nail, THA: total hip arthroplasty Below table 3
